# Molecular Cloning and Characterization of Carbonic Anhydrase XII from Pufferfish (*Takifugu rubripes*)

**DOI:** 10.3390/ijms19030842

**Published:** 2018-03-13

**Authors:** Kanij Rukshana Sumi, Soo Cheol Kim, Jewel Howlader, Won Kyo Lee, Kap Seong Choi, Hoy-Taek Kim, Jong-In Park, Ill-Sup Nou, Kang Hee Kho

**Affiliations:** 1 Department of Fisheries Science, College of Fisheries and Ocean Sciences, Chonnam National University, 50, Daehak-ro, Yeosu, Jeonnam 59626, Korea; krsumi@pstu.ac.bd (K.R.S.); wklee196@jnu.ac.kr (W.K.L.); 2 Department of Biomedical and Electronic Engineering, College of Engineering, Chonnam National University, Yeosu, Jeonnam 59626, Korea; sherlock0712@gmail.com; 3 Department of Horticulture, College of Life Science and Natural Resources, Sunchon National University, 255, Jungang-ro, Suncheon-Si, Jeollanam-do 57922, Korea; jewel.howlader81@gmail.com (J.H.); htkim@sunchon.ac.kr (H.-T.K.); jipark@sunchon.ac.kr (J.-I.P.); nis@sunchon.ac.kr (I.-S.N.); 4 Department of Food Science, College of Life Science and Natural Resources, Sunchon National University, 255, Jungang-ro, Suncheon-Si, Jeollanam-do 57922, Korea; chks@sunchon.ac.kr

**Keywords:** pufferfish, *Takifugu rubripes*, brain tissue, CA XII

## Abstract

In this study, an 1888-bp carbonic anhydrase XII (CA XII) sequence was cloned from the brain of the pufferfish, *Takifugu rubripes*. The cloned sequence contained a coding region of 1470-bp, which was predicted to translate into a protein of 490 amino acid residues. The predicted protein showed between 68–56% identity with the large yellow croaker (*Larimichthys crocea*), tilapia (*Oreochromis niloticus*), and Asian arowana (*Scleropages formosus*) CA XII proteins. It also exhibited 36% and 53% identity with human CA II and CA XII, respectively. The cloned sequence contained a 22 amino acid NH_2_-terminal signal sequence and three Asn-Xaa-Ser/Thr sequons, among which one was potentially glycosylated. Four cysteine residues were also identified (Cys-21, Cys-201, Cys-355, and Cys-358), two of which (Cys-21 and Cys-201) could potentially form a disulfide bond. A 22-amino acid COOH-terminal cytoplasmic tail containing a potential site for phosphorylation by protein kinase A was also found. The cloned sequence might be a transmembrane protein, as predicted from in silico and phylogenetic analyses. The active site analysis of the predicted protein showed that its active site residues were highly conserved with tilapia CA XII protein. Homology modeling of the pufferfish CA XII was done using the crystal structure of the extracellular domain of human carbonic anhydrase XII at 1.55 Å resolution as a template. Semi-quantitative reverse transcription (RT)-PCR, quantitative PCR (q-PCR), and in situ hybridization confirmed that pufferfish *CA XII* is highly expressed in the brain.

## 1. Introduction

Carbonic anhydrases (CAs) catalyze the reversible conversion of carbon dioxide and water to bicarbonate and proton in the following reaction: CO_2_ + H_2_O ⇄ HCO_3_^−^ + H^+^ [1]. CAs are involved in several physiological processes such as acid-base homeostasis, electrolyte secretion, ion transport, biosynthetic reactions, bone resorption, and tumorigenicity [2,3,4]. Moreover, many CA isoforms have been used as potential targets for the design of the functional pharmacological drugs, such as antiglaucoma, anticonvulsant, antiurolithic, and for the treatment of obesity [5]. Thirteen catalytically active CAs (cytosolic: CAs I, II, III, VII, and XIII; membrane-associated: CAs IV, IX, XII, XIV, and XV; mitochondrial: CAs VA and VB; secreted CA VI); and three inactive CAs (CA VIII, X, and XI), all belonging to the α-CA gene family, are found in the animal kingdom [4,6]. This study focuses on CA XII found in pufferfish (*Takifugu rubripes*).

CA XII is a membrane-bound anhydrase that was independently isolated and characterized from human cells by Ivanov et al. [7] and Türeci et al. [8]. CA XII is a type I transmembrane protein and has three zinc-binding histidine residues in its active extracellular domain. Thus, it resembles other active CAs. It also has two potential sites for asparagine glycosylation. CA XII shows 30–42% sequence identity with other CAs [8]. The presence of an NH_2_-terminal signal peptide and a short COOH-terminal cytoplasmic tail is a characteristic feature of human CA XII [8]. The crystal structure of human CA XII has been described at a 1.55 Å resolution [9].

In humans, CA XII is expressed in many tissues, including the aorta, bladder, brain, colon, esophagus, kidneys, liver, lungs, lymph nodes, mammary glands, ovaries, prostate, pancreas, peripheral blood lymphocytes, rectum, stomach, skeletal muscles, skin, spleen, testis, trachea, and uterus [7,8,10]. Halmi [11] reported that the expression of mammalian CA XII is upregulated in some cancers and that its expression might be induced by hypoxia.

Although the GenBank database of the National Center for Biotechnology Information (NCBI) documents the sequences of CA XII in the genomes of many fish species, such predictions are only based on gene homology and no published literature describes the isolation and characterization of CA XII in fish. We used the information available on the NCBI platform to isolate and molecularly characterize the gene for the CA XII from pufferfish (*T. rubripes*).

## 2. Results

### 2.1. Molecular Cloning and Characterization of CA XII from Pufferfish

**Figure 1:**
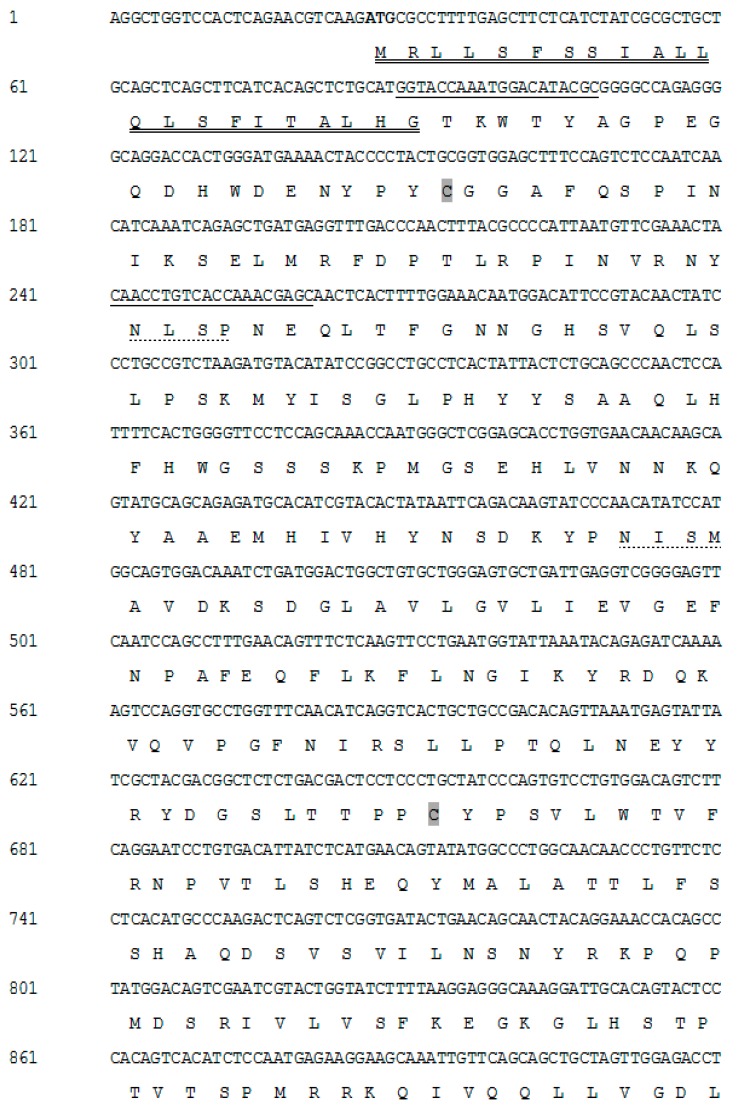

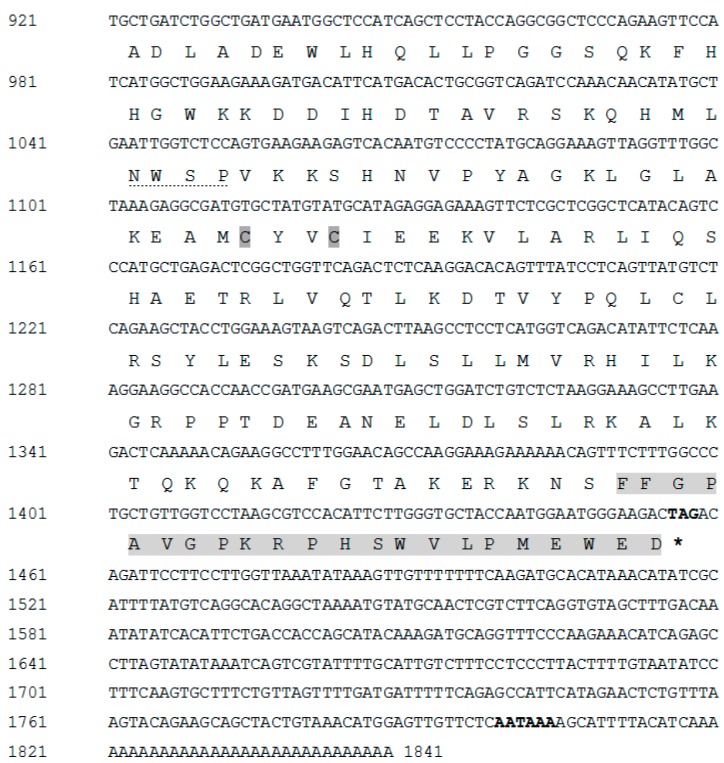
The nucleotide and deduced amino acid sequences of pufferfish CA XII. The deduced amino acid sequence is reported in one-letter code. The start, stop codon (asterisks), and the potential polyadenylation signal are in bold font. The regions used for designing primers for RT-PCR and q-PCR are underlined in the sequence. The N-terminal signal peptide is double underlined. The potential glycosylation site is underlined with a dotted line. The four cysteine residues (Cys-21, Cys-201, Cys-355, and Cys-358) are highlighted in grey; two of them (Cys-21, Cys-201) likely form a disulfide bond. The C-terminal cytoplasmic tail is highlighted in light grey.

The NCBI CA XII sequence of *T. rubripes* was used for molecular cloning of CA from pufferfish. The conformance of the cloned product to the 1888-bp nucleotide sequence (GenBank accession no. MF134897) was checked by 5′ and 3′-rapid amplification of cDNA ends (RACE)-PCR. The 1888-bp transcript obtained upon RACE PCR contained an open reading frame (ORF) of 1470-bp, including a 25-bp 5′-untranslated region (UTR) and a 390-bp 3′-UTR (Figure 1).

Although little difference was observed between the NCBI CA XII and the cloned CA sequences, two regions (46-bp 5′-UTR and 342-bp 3′-UTR) were found to be unique to the NCBI CA XII sequence. Moreover, in the cloned sequence, an adenine/guanine substitution was observed at the 1649 position in the 3′-UTR. The deduced amino acid sequence of 490 residues from ORF showed that the cloned CA protein from pufferfish exhibited 36% and 53% identity with human CA II and CA XII proteins, respectively. It also exhibited 68% identity with large yellow croaker (*Larimichthys crocea*), 65% identity with tilapia (*Oreochromis niloticus*), and 56% identity with Asian arowana (*Scleropages formosus*) CA XII proteins.

A 22-amino acid NH_2_-terminal signal sequence was predicted to be present in the cloned CA sequence (Figure 1). Three potential Asn-Xaa-Ser/Thr sequons were also observed in the sequence. One located at position 149 potentially served as an N-linked glycosylation site, with a probability of over 0.5. Moreover, four cysteine residues (Cys-21, Cys-201, Cys-355, and Cys-358) were also identified in the cloned CA sequence (Figure 1). Two of these cysteine residues (Cys-21 and Cys-201) likely formed a disulfide bond based on folding (Figure S1).

A 22 amino acid COOH-terminal cytoplasmic tail that contained a potential site for phosphorylation by protein kinase A was also found. The predicted molecular mass of the cloned CA sequence was 55.64 kDa and it was predicted to be inserted into the plasma membrane.

**Figure 2:**
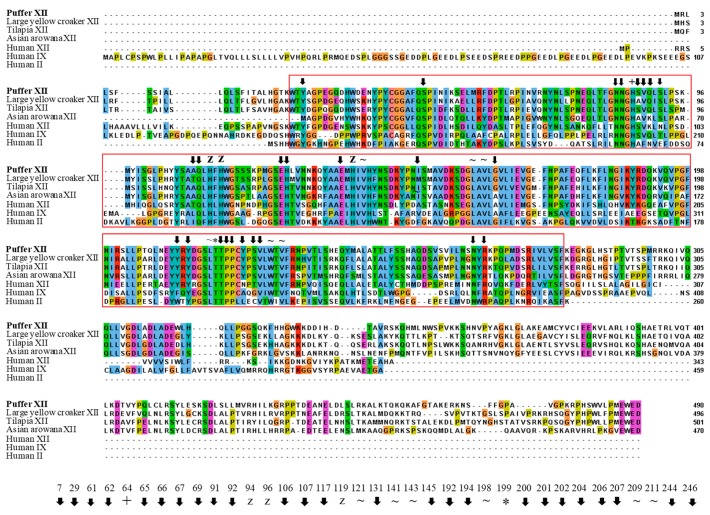
Comparison of putative active sites in CA II, CA IX, and CA XII sequences derived from human and several species of fish. The CA IX and XII sequences were aligned against human CA II (GenBank accession no. NP_000058.1) by Clustal Omega to identify putative active sites. The color was given according to default color scheme used for alignments in Clustal X, where blue: hydrophobic residues; red: positive charge residues; magenta: negative charge residues; green: polar residues; pink: cysteines; orange: glycines; yellow: prolines; cyan: aromatic residues; white: unconserved residues. Numbers below the alignment represent the positions in the active site relative to the human CA II reference sequence [12]. Notations full-forms are as follows: 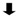: putative active site pockets; z: zinc binding site; +: proton shuttling site; ~: substrate associated pocket; *: Thr-199 loop site. The red colored box represents the potential carbonic anhydrase domain of pufferfish CA XII. The pufferfish CA XII cloned in the present study is highlighted in bold font. GenBank accession numbers for the sequences are as follows: CA IX (human, NP_001207.2); CA XII (human, NP_996808.1; large yellow croaker, KKF18125.1; tilapia, XP_005470754.1); CA XII-like sequence (Asian arowana, KPP78156.1).

The amino acid residues in the active sites of CA proteins in human and fish are highly conserved (Figure 2). The cloned CA protein had 28 and 30 amino acid residues identical with the putative active site pockets of human CA II and human CA XII proteins, respectively. In pufferfish CA XII, substitutions were observed at positions 65, 66, 67, 69, 91, 204, and 206 when compared with human CA II. Moreover, 100% similarity of active site residues was observed between pufferfish and tilapia CA XII proteins, while only two and four amino acid residues differed between the pufferfish CA XII and the large yellow croaker CA XII and Asian arowana CA XII, respectively.

**Figure 3:**
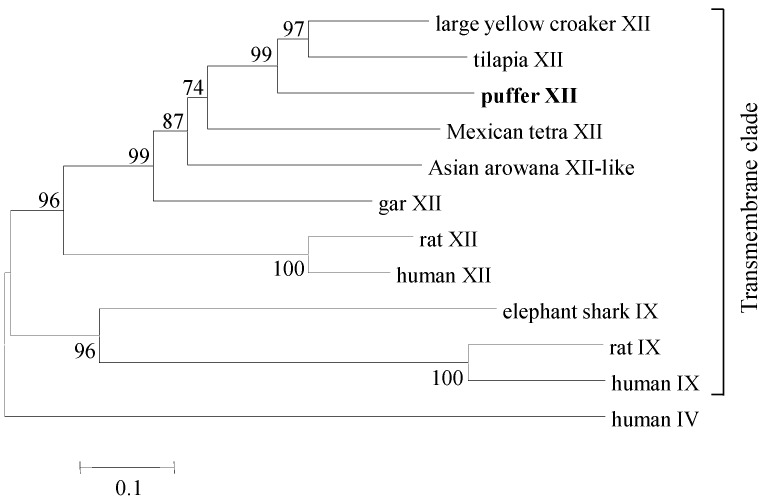
Phylogenetic analysis of carbonic anhydrase (CA) proteins present in mammalian (human, *Homo sapiens*; rat, *Rattus norvegicus*) and several fish species (pufferfish, *Takifugu rubripes*; tilapia, *Oreochromis niloticus*; large yellow croaker, *Larimichthys crocea*; gar, *Lepisosteus oculatus*; Asian arowana, *Scleropages formosus*; Mexican tetra, *Astyanax mexicanus*; elephant shark, *Callorhinchus milii*). The pufferfish CA XII cloned in the present study is highlighted in bold font. The phylogenetic tree was constructed using the neighbor-joining method. The results were confirmed by bootstrapping. Values given at the nodes indicate bootstrap percentages for the given split among 1000 repetitions. GenBank accession numbers for the sequences used to generate the tree are as follows: CA IV (human, NP_000708.1); CA IX (human, NP_001207.2; rat, XP_008761898.1; elephant shark, XP_007895370.1); CA XII (human, NP_996808.1; rat, XP_006243372.1; large yellow croaker, KKF18125.1; tilapia, XP_005470754.1; Mexican tetra, XP_007228316.1; gar, XP_015199103.1); CA XII-like sequence (Asian arowana, KPP78156.1).

Several clades were formed during phylogenetic analysis of the broad class of transmembrane CAs. Mammalian CA IX and XII formed one monophyletic clade, whereas all CA XII from fish formed a separate clade. The human CA IV was used as a monophyletic outgroup (Figure 3). The fish clade showed divergence with evolution over time. The gar CA XII showed the earliest divergence, while the pufferfish CA XII grouped more closely with the tilapia CA XII and large yellow croaker CA XII (Figure 3).

**Figure 4:**
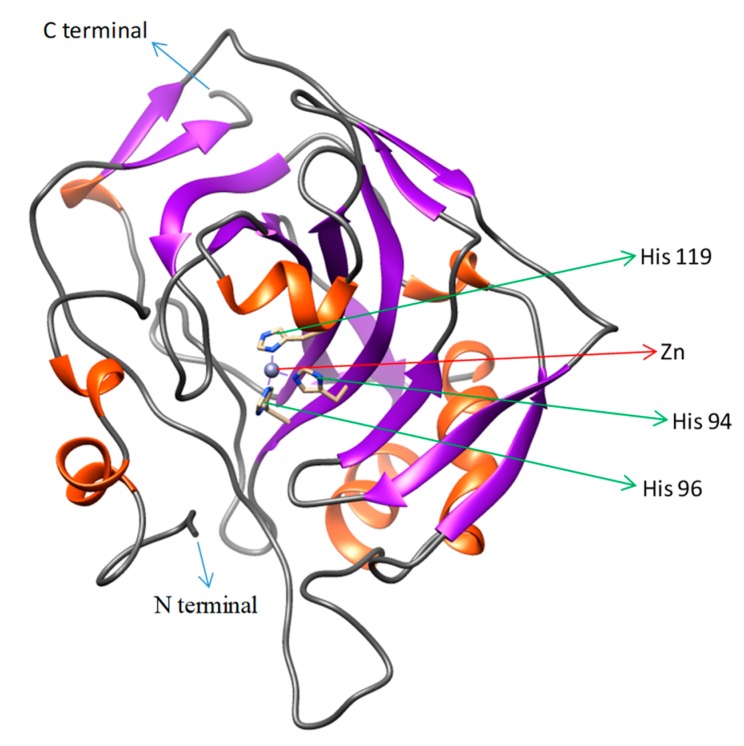
Three-dimensional model of CA XII isolated from pufferfish (*Takifugu rubripes*). The N- and C-termini are marked with blue arrows. The zinc ion in the active site and coordinated histidine residues are marked in figure with red and green arrows, respectively. The model was generated using Chimera and the domains between the N- and C-termini are predicted from the secondary structure.

The structure of the 490 amino acid sequence of the cloned CA XII from pufferfish was predicted by homology modeling. The template for modeling was selected by BLAST searches of NCBI (available online: https://blast.ncbi.nlm.nih.gov/Blast.cgi) and the protein structure databases (available online: http://www.rcsb.org). Crystal structure of the extracellular domain of the human carbonic anhydrase XII (PDB ID: 1JCZ) was chosen as the template because of high identity (53%) and low *E*-value (9 × 10^−103^). The UCSF Chimera Modeller interface was used for modeling. The model was built by assuming co-crystallization of the zinc ion (Zn^2+^) ligand. The modeling program conforms the cloned CA XII sequence onto the template sequence. Based on different statistical scores (GA341 and zDOPE), the best model of CA XII was selected. To further optimize the model, stereoscopic properties were validated with the ProQ, Verify3D, and ERRAT tools. The evaluation results for the CA XII model were as follows. ProQ, LGscore: 5.873 (value >4 implies an extremely good model) and MaxSub: 0.585 (values >0.5–<0.8 implies a very good model); Verify3D: 3D/1D profile score: 92.49%; ERRAT overall quality factor: 94%. These results confirmed that the predicted CA XII assigned favorable positions to each amino acid residue. The predicted 3D structure of pufferfish CA XII is presented in Figure 4. The figure was generated using Chimera, with different and colored motifs denoting the standard secondary structure domains. The active site Zn^2+^ and coordinated histidine residues are also highlighted.

### 2.2. Expression Analysis of Pufferfish CA XII

**Figure 5:**
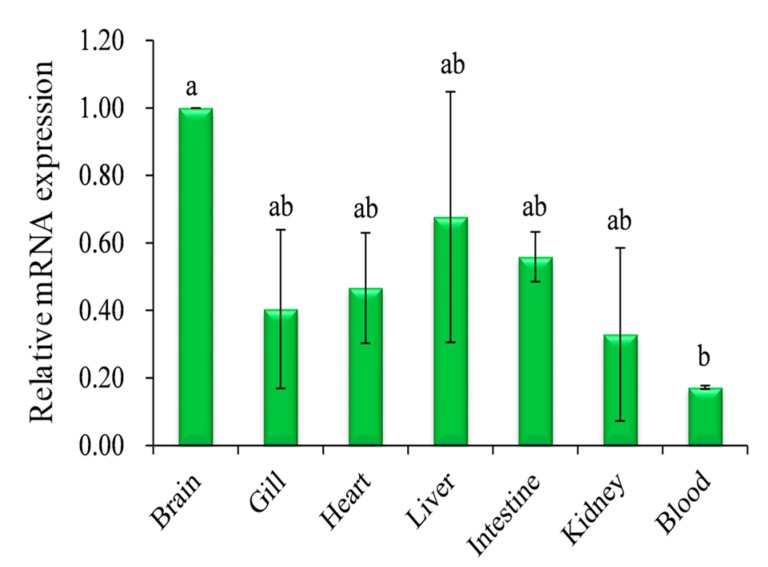
Relative mRNA expression (means ± SD, *N* = 3) of *CA XII* in pufferfish (*Takifugu rubripes*) tissues as determined by quantitative PCR. Expression in all tissues is normalized to expression in the brain. The expressions with the different letters are significantly (*p <* 0.05) different.

Relative mRNA expression was quantified for major tissues by q-PCR (Figure 5). Significantly high (*p* < 0.05) expression was observed in the brain compared to other tissues, but there were no statistical differences in the expression of *CA XII* among gills, heart, liver, intestine, and kidney (Figure 5). Significantly low (*p <* 0.05) expression was found in the blood. Supporting data were also obtained from semi-quantitative RT-PCR expression analysis (Figure S2).

**Figure 6:**
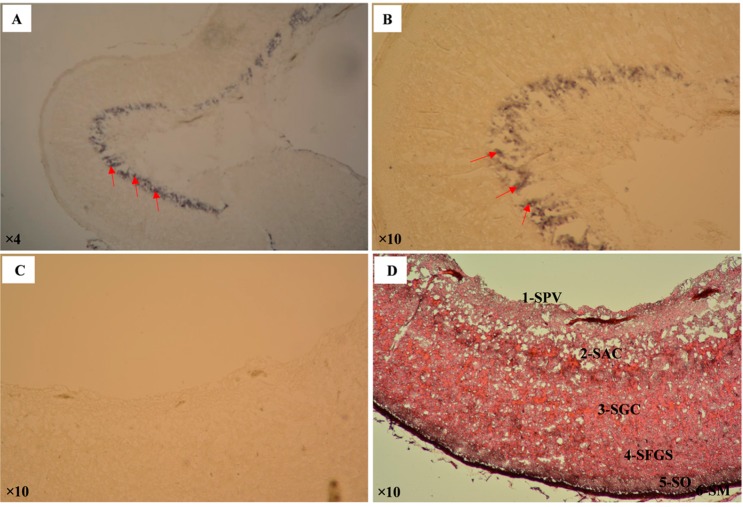
Light micrographs of pufferfish brain where *CA XII* mRNA expression is localized to the optic tectum by in situ hybridization. The hybridization of the *CA XII* mRNA probe is seen as purple signals in panels (**A**,**B**) (red arrowheads); Panel (**C**) represents the negative control for in situ hybridization of the mRNA probe; Panel (**D**) shows hematoxylin and eosin staining of the optic tectum with the following structures: (1) Stratum (Str.) periventriculare (SPV) in the inner region, (2) Str. album centrale (SAC), followed by (3) Str. griseum centrale (SGC), (4) Str. fibrosum et grisium superficiale (SFGS), (5) Str. opticum (SO), and (6) Str. marginale (SM).

For detection of *CA XII* mRNA expression in pufferfish optic tectum, in situ hybridization was performed with brain sections. The presence of purple color meant a positive hybridization signal (Figure 6A,B). No color developed in the negative controls (Figure 6C). The positive signal was localized to the stratum album centrale of the optic tectum, as revealed by hematoxylin and eosin staining (Figure 6D).

## 3. Discussion

CA XII in mammals are well characterized. Recent study also investigated the inhibition of human CA XII with benzothiazole-based sulfonamides [13], but to date no reports on the cloning and characterization of CA XII from fish have been published. NCBI provides the sequences of CA XII of many fish species. Based on this information, we endeavored to clone the CA XII from pufferfish. The cloned sequence showed 36% similarity with the human CA II and 53% similarity with the human CA XII protein. It also showed 56–68% similarity with other fish CA XII proteins. Türeci et al. [8] reported that type I transmembrane proteins contain a 29 amino acid signal peptide. They also identified a 29 amino acid cytoplasmic tail in the human CA XII protein. In contrast to the cloned pufferfish sequence, we found a 22 amino acid signal sequence and a 22 amino acid cytoplasmic tail. Moreover, the cloned sequence was discovered to have one N-linked glycosylated site and four cysteine residues (Cys-21, Cys-201, Cys-355, and Cys-358). Among the latter, two cysteine residues, Cys-21 and Cys-201, are predicted to form a disulfide bond (Figure S1). Multiple sequence alignment revealed that the active site residues of CA proteins are highly conserved among vertebrates (Figure 2), especially the active site residues of pufferfish CA XII and tilapia CA XII protein. 

Extracellular CAs can be secreted (CA VI; [14]), transmembrane (CA IX, XII, and XIV; [8,15,16,17,18]), and glycosylphosphatidylinositol (GPI)-anchored (CA IV and XV; [19,20,21]). Our results are consistent with this grouping. In the phylogenetic tree that we constructed with a few select extracellular CAs, the mammalian transmembrane proteins CA IX and CA XII, and fish CA XII proteins formed one broad clade, whereas the GPI-anchored, human CA IV, formed a divergent clade (Figure 3). Our tree predicted CA XII emergence to have taken place after the evolution of CA IV and CA IX. This was also reported by Esbaugh and Tufts [22] in a phylogenetic analysis of CA proteins.

Homology modeling of the cloned puffer CA XII was done using a 1.55 Å resolution crystal structure of the extracellular domain of the human CA XII as a template [9]. The cloned puffer CA XII sequence and the template sequence were aligned with Multi Align Viewer to identify the motifs and residues conserved between them [23]. Co-crystallization of the Zn^2+^ ligand was given special consideration during template selection because Zn-bound hydroxide ions play an important role in catalysis by CA proteins [1].

*CA XII* mRNA was expressed in the brain, gills, heart, liver, intestine, kidney, and blood of pufferfish, with the brain as the site of highest expression. Previous studies have also found that *CA XII* is expressed in a wide spectrum of normal tissues [7,8,10,24,25]. For example, mRNA expression of *CA XII* has been reported in several human tissues, including the aorta, bladder, brain, colon, esophagus, kidneys, liver, lungs, lymph node, mammary glands, ovary, prostate, pancreas, peripheral blood lymphocytes, rectum, stomach, skeletal muscles, skin, spleen, testes, trachea, and uterus [7,8,10].

Upon in situ hybridization, mRNA expression of pufferfish *CA XII* gene was detected in the optic tectum of the brain (Figure 6A,B). Hematoxylin and eosin staining of the optic tectum showed that expression was localized to the stratum (str.) periventriculare (SPV) in the inner region, str. album centrale (SAC), str. griseum centrale (SGC), str. fibrosum et griseum superficiale (SFGS), str. opticum (SO), and str. marginale (SM; Figure 6D), as also reported by Mishra and Devi [26]. In vertebrates, the optic tectum represents a major portion of the midbrain. In fish as well as some non-mammalian vertebrates, the optic tectum represents one of the largest parts of the brain. The primary roles of the tectum are visual processing and control of eye movement. The detection of pufferfish *CA XII* in the stratum album centrale of the optic tectum indicates that it might control regional ion and fluid movement in the central nervous system, as reported previously by Trachtenberg and Sapirstein [27].

Ivanov et al. [28], Wykoff et al. [29], and Watson et al. [30] posited that *CA XII* expression is induced by hypoxia because it is upregulated in certain tumors. These authors also suggested that CA XII may be an excellent marker for hypoxia in tumors. The identification of CA XII in pufferfish could allow the development of reagents to identify cancer cells and aid in the development of potential therapeutic drugs.

## 4. Materials and Methods

### 4.1. Experimental Fish

The fish were collected from Chungnam Prefectural Fisheries Institute, Daejeon, Korea, and transported to the Department of Fisheries Science, Chonnam National University, Korea. The approval of experimental animals (Approval No.: CNU IACUC-YS-2015-6, Approval date: 22 December 2015) was done by the University of Chonnam National Animal Care and Use Committee and all animal experimentation were performed according to the Guide for the Care and Use of Laboratory Animals of the National Institutes of Health.

The fish were then euthanized using an overdose of ethyl 3-aminobenzoate methanesulfonate (MS-222; 1 g/L; Sigma-Aldrich, St. Louis, MO, USA). Following cardiac puncture, blood samples were collected in 250 IU heparinized saline (NaCl, 280 mM; KCl, 6 mM; CaCl_2_, 5 mM; MgCl_2_, 3 mM; NaSO_4_, 0.5 mM; NaHPO_4_, 1 mM; NaHCO_3_, 8 mM; urea, 350 mM; glucose, 5 mM; and trimethylamine oxide, 70 mM; [31]), snap frozen in liquid nitrogen, and stored at −80 °C. The brain, gills, heart, liver, intestine, and kidney were excised, snap frozen in liquid nitrogen, and stored at −80 °C until further use.

For cryosection preparation, the brain was washed with phosphate-buffered saline (PBS; pH 7.4) and immersion fixed in 4% paraformaldehyde (PFA) overnight. The fixed tissue was washed three times with PBS for 1 min each time. The tissue was then suspended in 30% sucrose and kept at 4 °C overnight. The tissue was embedded in Shandon Cryomatrix medium (Thermo Fisher Scientific, Waltham, MA, USA), kept at room temperature for 1 h, and subsequently stored at −20 °C until further use. Sectioning of the frozen brain was done using a cryostat (CM 3050; LEICA, Wetzlar, Germany). Thin (10 µm) sections were collected onto electrostatically charged slides (SuperFrost Plus; VWR International, Radnor, PA, USA), air-dried for 30 min, and then stored at −20 °C until further use.

### 4.2. RNA Extraction, RT-PCR, and Molecular Cloning of CA XII

An RNeasy mini kit (Qiagen, Hilden, Germany) was used to extract the total RNA from pufferfish tissues (brain, gills, heart, liver, intestine, kidney, and whole blood cells). The samples were treated with RNase-free DNase (Promega, Madison, WI, USA) during the extraction procedure to remove any genomic DNA. The total RNA was quantified by spectrophotometry on a NanoDrop^®^ NP-1000 apparatus (Thermo Fisher Scientific). From each tissue sample, 1 µg of total RNA was reverse transcribed into complementary DNA (cDNA) by Superscript^®^ III First-Strand synthesis kit (Invitrogen, Carlsbad, CA, USA) following the manufacturer’s instructions.

Reverse transcription PCR (RT-PCR) was performed using primer pairs designed against the CA XII sequence in NCBI (XM_011607386.1); the sense strand sequence: 5′-GTCAAGATGCGCCTTTTGAG-3′, and the antisense strand sequence: 5′-CTCCATGTTTACAGTAGCTG-3′. RT-PCR was carried out with Phusion^®^ High-Fidelity DNA Polymerase (New England Biolabs, Piswich, MA, USA) in a 20 µL reaction volume: 1 µL cDNA template from tissue + 1 µL (20 pmol) each of sense and antisense primer + 4 µL of 5× Phusion HF buffer + 2 µL of dNTP (200 µM) + 0.5 µL of 1 U Phusion DNA polymerase + 10.5 µL of sterilized distilled water (dH_2_O). The thermal cycler program for PCR amplification was as follows: 95 °C for 2 min (initial denaturation); 35 cycles at 95 °C for 45 s, 60 °C for 45 s (annealing) and 72 °C for 1 min (extension); 72 °C for 5 min (final extension). The amplified PCR products were separated on 1% agarose gel and purified using a gel extraction kit (Promega, Madison, WI, USA).The purified PCR products were ligated into the pTOP Blunt V2 vector (Enzynomics, Daejeon, Korea) and transformed into *Escherichia coli* DH5α competent cells (Enzynomics, Daejeon, Korea). Later, plasmid DNA was extracted from the transformed cells with a plasmid miniprep kit (Qiagen, Hilden, Germany) and sequenced on a Macrogen Online Sequencing System (Macrogen, Seoul, Korea).

### 4.3. Rapid Amplification of 5′and 3′ cDNA Ends

Rapid amplification of 5′ and 3′ cDNA ends (RACE) was carried out with SMARTer^®^ RACE 5′/3′ kit (Clontech Laboratories, Inc., Mountain View, CA, USA) according to the user manual. Gene-specific primers derived from the initial primers were used for cloning by the addition of a 15-bp (GATTACGCCAAGCTT) sequence to the 5′-end of both gene-specific primer sequences; antisense primer: 5′-GATTACGCCAAGCTTGCTGTGGTTTCCTGTAGTTGCTGTTCAG-3′ and sense primer: 5′-GATTACGCCAAGCTTCTGAACAGCAACTACAGGAAACCACAGC-3′. RACE PCR reactions were performed with 2.5 µL of RACE ready cDNA, 5 µL of 10× universal primer mix (UPM, 5′-CTAATACGACTCACTATAGGGCAAGCAGTGGTATCAACGCAGAGT-3′), 1µL of gene-specific primers (10 pmol), and SeqAmp DNA Polymerase in a total volume of 50 µL. Touchdown PCR was performed on the reaction mixture for 25 cycles, according to kit instructions. Five additional cycles were performed for 5′-RACE to enable band detection. Purification of RACE PCR products was done using NucleoSpin^®^ Gel and PCR Clean-Up kit (Clontech Laboratories, Inc., Mountain View, CA, USA). The purified RACE products were ligated into linearized pRACE vector (Clontech Laboratories, Inc., Mountain View, CA, USA) and transformed into Stellar Competent Cells supplied with the SMARTer^®^ RACE 5′/3′ kit and later sequenced at Macrogen Online Sequencing System (Macrogen, Seoul, Korea). The fullness of the length of the sequence was confirmed by matching the RACE products with the initially cloned cDNA fragments.

### 4.4. Sequence Analysis

To analyze the pufferfish CA XII sequence, different online software programs were used. Basic Local Alignment Search Tool (BLASTP) was used to ascertain the homology of pufferfish CA XII protein to the CA XII of other species in the NCBI database. To predict the presence of N-terminal signal peptide and the bonding state of cysteines in the protein sequence, SignalP 4.1 (available online: www.cbs.dtu.dk/services/SignalP/) and CYSPRED [32] were used, respectively. Physical and chemical parameters associated with the primary sequence of the protein were calculated using ProtParam (available online: http://expasy.org/tools/protparam.html). Determination of the protein location within cells was done with Protcomp (available online: http://linux1.softberry.com/berry.phtml). The CA domain within the CA XII protein of pufferfish was identified using the SMART web tool from the European Molecular Biology Laboratories (EMBL; available online: smart.embl-heidelberg.de). Multiple sequence alignment of CA proteins was accomplished with Clustal Omega [33,34]. We used Jalview, version 2.10.0 (available online: www.jalview.org) [35] for editing and visualizing the aligned sequences.

### 4.5. Phylogenetic Analysis

To construct a phylogenetic tree, CA XII sequences from different fish species along with mammalian CA IX and CA XII sequences were retrieved from the NCBI database using the BLASTP algorithm. The human CA IV sequence was used as a monophyletic outgroup. The retrieved protein sequences were aligned using Clustal Omega [33,34]. The tree was generated with MEGA (version 6.06) using the neighbor-joining algorithm [36,37]. The split at each node of the tree was confirmed by bootstrapping 1000 replicates (corresponding values indicated in Figure 3). The GenBank accession numbers of the CA sequences for constructing the phylogenetic tree are given in the legend of Figure 3.

### 4.6. Template Identification and Homology Modeling of CA XII in Pufferfish

The crystal structure of the extracellular domain of human CA XII (accession No. 1JCZ), at a resolution of 1.55 Å, was chosen as the template for three-dimensional (3D) homology modeling of pufferfish CA XII protein by MODELLER (available online: https://salilab.org/modeller/). The model is generated by satisfying spatial restraints [38]. The template for modeling was selected by considering the identities and *E*-values of all the hits obtained against pufferfish CA XII sequence in Protein Data Bank (available online: www.rcsb.org) [39]. The Multi Align Viewer tool was used to align pufferfish CA XII with the template followed by homology modeling with MODELLER using UCSF Chimera. The best model of CA XII was chosen out of the five generated, based on their normalized Discrete Optimized Protein Energy statistical scores (zDOPE). Multi RCSB (Research Collaborators for Structural Bioinformatics) servers (available online: http://deposit.pdb.org/validate/) were used to validate the structure. ProQ was used to estimate structural features of the model (available online: http://www.sbc.su.se/~bjornw/ProQ/ProQ.html). Verify3D (available online: http://nihserver.mbi.ucla.edu/Verify3D/) was used to confirm the compatibility of the 3D atomic model with its own amino acid sequence [40]. ERRAT (available online: http://services.mbi.ucla.edu/ERRAT) was also used for this process. Visualization of the model structure was done with Chimera (available online: https://www.cgl.ucsf.edu/chimera/).

### 4.7. Semi-Quantitative RT-PCR Analysis of Expression

For semi-quantitative PCR, gene-specific primers (forward: 5′-GGTACCAAATGGACATACGC-3′ and reverse: 5′-GCTCGTTTGGTGACAGGTTG-3′; Figure 1) were used to amplify the pufferfish *CA XII* and *T. rubripes β-actin* (XM_003964421.2; forward primer: 5′-TACAGGTCCTTACGGATGTC-3′ and reverse primer: 5′-CGTGGGTACTCCTTCACTAC-3′) was used as a normalization control. The reaction mixtures comprised 1 µL cDNA template each from the brain, gills, heart, liver, intestine, kidney, and blood; 1 µL (20 pmol) each of forward and reverse primer; 10 µL of Prime Taq Premix (2×; GENETBIO); and 7 µL of sterilized distilled water (dH_2_O). The final volume was 20 µL. The amplification conditions for PCR were the same as described earlier.

### 4.8. Quantitative PCR

Quantitative PCR (q-PCR) was performed in the LightCycler^®^ 96 System (Roche, Germany) using 2× qPCRBIO SyGreen Mix Lo-Rox (PCR Biosystems Ltd., London, UK). The gene-specific primers and *β-actin* primers used for q-PCR were the same as those used for semi-quantitative PCR. The reaction mixture comprised 1 µL of cDNA template, 1 µL (10 pmol) each of forward and reverse primer, 10 µL of SyGreen Mix, and 7 µL of dH_2_O in a total volume of 20 µL. The programmed PCR conditions were: pre-incubation at 95 °C for 5 min; amplification for 40 cycles (95 °C for 30 s, 60 °C for 30 s, and 72 °C for 30 s); the melting temperature was defined by the system. Relative gene expression was calculated by the 2^−∆∆*C*T^ method [41].

### 4.9. In Situ Hybridization

A pair of primers (forward: 5′-GTTCAGACTCTCAAGGACAC-3′ and reverse: 5′-GACAATGCAAAATACGACTG-3′) were used to produce a 495-bp product, which was used in turn as an antisense riboprobe for in situ hybridization. The amplified product was cloned into the pTOP Blunt V2 vector (Enzynomics, Daejeon, Korea) and sequenced to confirm its sequence and orientation. After purification, the product was suspended in 5 μL of RNase-free H_2_O. Probe-labeling was performed with T7 RNA polymerase and digoxigenin (DIG) RNA labeling mix (Roche, Mannheim, Germany) according to the kit manual.

Hybridization buffer (5 mL deionized formamide, 2.5 mL of 20× saline sodium citrate [SSC], 100 μL of 0.1% Tween-20, 92 μL of 1 M citric acid (pH 6.0), and diethyl pyrocarbonate (DEPC)-H_2_O up to 20 mL total volume) and yeast total RNA (50 μL) were used to pre-hybridize the brain tissue for 2 h. The brain tissue was hybridized with the RNA probe at 65 °C overnight. The hybridized brain sections were washed in the following sequence: 75% hybridization mix plus 25% 2× SSC (10 min, 65 °C), 50% hybridization mix plus 50% 2× SSC (10 min, 65 °C), 25% hybridization mix plus 75% 2× SSC (10 min, 65 °C), 0.2× SSC (twice, 30 min), 75% 0.2× SSC plus 25% PBS with Tween-20 (PBST; 5 min, room temperature), 50% 0.2× SSC plus 50% PBST (5 min, room temperature), 25% 0.2× SSC plus 75% PBST (5 min, room temperature), and PBST (5 min, room temperature).

The tissue sections were first treated with a blocking solution (10% calf serum in PBST) for 1 h at room temperature and then treated overnight at −20 °C with an alkaline phosphatase-conjugated anti-digoxigenin antibody (diluted 1:2000 in blocking solution [Roche]) to detect the hybridization signal. The tissue was washed six times for 15 min each time in PBST at room temperature and then rinsed with alkaline Tris buffer (1 M Tris at pH 9.5, 1 M MgCl_2_, 5 M NaCl, and 10% Tween-20; three washes, 5-min washes at room temperature). For color visualization, the tissue was sprayed with a mixture (nitroblue tetrazolium and 5-bromo-4-chloro-3-indolyl phosphate tablets dissolved in 10 mL H_2_O). Finally, the sections were kept in a dim and moist chamber for 5 h to allow the color to develop. After color development, the sections were washed with PBST, fixing with 4% PFA for 1 h, mounting with Aquamount (Aqua Polymount, Polisciences Inc., Warrington, PA, USA), and finally cover-slipped. Images were captured under a SMZ1500 stereo microscope (Nikon, Tokyo, Japan). For testing the specificity of hybridization, the probe was removed from the hybridization buffer.

### 4.10. Statistical Analyses

Statistical analyses were performed to detect significant changes in *CA XII* expression in different tissues of pufferfish using one way Analysis of Variance (ANOVA). Tukey’s test was performed to compare the mean mRNA expression in different tissues relative to the brain. Statistical Analysis System (Version 16.0; SPSS, Chicago, IL, USA) was used for all analyses.

## 5. Conclusions

In conclusion, an 1888-bp sequence was cloned and confirmed by RACE-PCR from the pufferfish (*T. rubripes*) brain, based on its genome sequence available on NCBI. A BLAST search indicated that the 490 amino acid translated protein shows 56–68% identity with large yellow croaker, tilapia, and Asian arowana CA XII proteins. It also exhibits 36% and 53% identity with human CA II and CA XII, respectively. From *in silico* and phylogenetic analyses, it can be inferred that the cloned protein might be a transmembrane CA XII protein. Similar conclusions can also be made based on the high similarity of pufferfish CA XII active site residues with tilapia CA XII active site residues. Homology modeling was also performed on the cloned pufferfish CA XII sequence. Expression of pufferfish *CA XII* was observed in all organs tested, with the highest expression occurring in the brain. In situ hybridization also confirmed pufferfish *CA XII* expression in brain tissue sections.

## Supplementary Materials

Supplementary materials can be found at http://www.mdpi.com/1422-0067/19/3/842/s1.
