# Supplementary material for: Molecular Cloning and Characterization of Carbonic Anhydrase XII from Pufferfish (Takifugu rubripes)
Source: Int J Mol Sci. 2018 Mar 13;19(3):842. doi: 10.3390/ijms19030842 (PMC5877703; doi:10.3390/ijms19030842)
Supplement: Supplementary file 1 [file ijms-19-00842-s001.pdf]

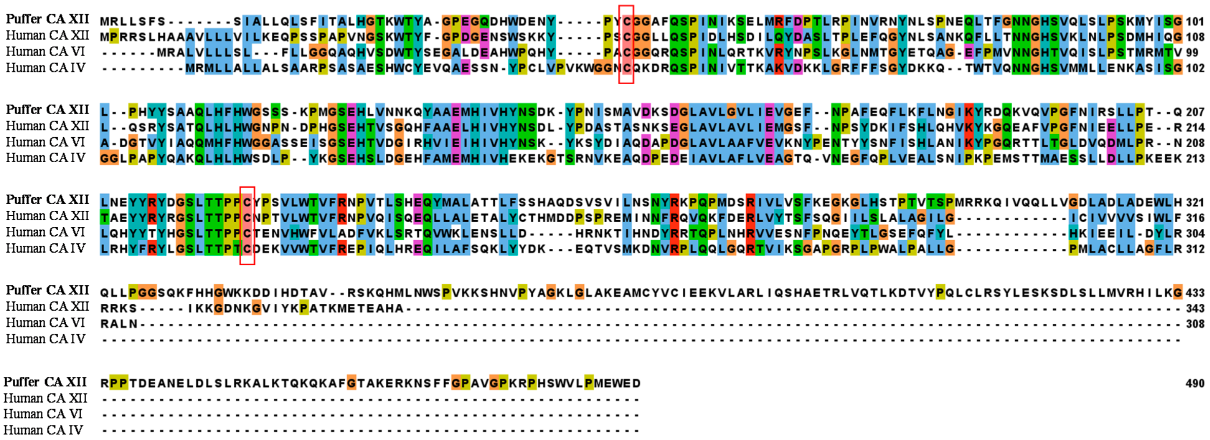

Figure S1. Multiple sequence alignment of membrane-bound carbonic anhydrase proteins: pufferfish CA XII, human CA XII, human CA VI, and human CA IV. The red colored hollow boxes show the conserved cysteine residues that likely form a disulfide bond in the CA proteins.

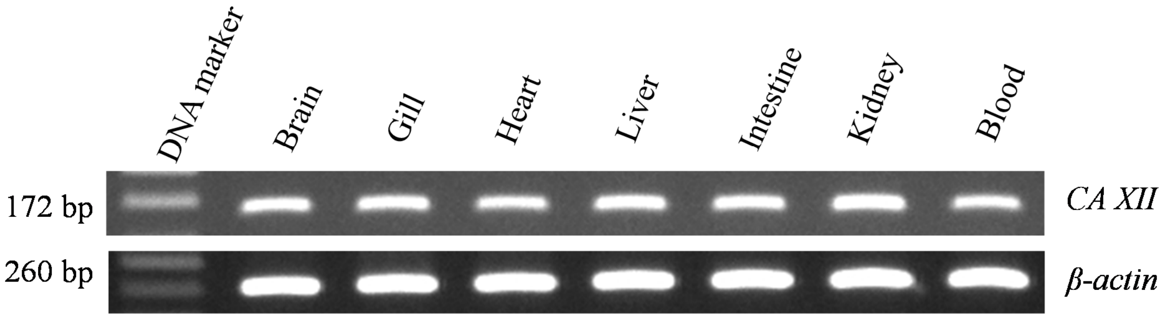

Figure S2. mRNA expression of carbonic anhydrase *CA XII* in different tissues (brain, gill, heart, liver, intestine, kidney and blood) of pufferfish (*Takifugu rubripes*) quantified by reverse transcription polymerase chain reaction (RT-PCR). *β-actin* was used as internal control.
